# Carbon Dots Meet MRI: Metal Doping for a Smart Contrast Agent Design

**DOI:** 10.3390/ijms27031436

**Published:** 2026-01-31

**Authors:** Oana Elena Carp, Cristina Mariana Uritu, Adina Coroaba, Silviu-Iulian Filipiuc, Conchi O. Ania, Narcisa Laura Marangoci, Mariana Pinteala

**Affiliations:** 1 Centre of Advanced Research in Bionanoconjugates and Biopolymers, “Petru Poni” Institute of Macromolecular Chemistry, 700487 Iasi, Romaniaadina.coroaba@icmpp.ro (A.C.); silviu.filipiuc@umfiasi.ro (S.-I.F.); conchi.ania@cnrs-orleans.fr (C.O.A.); nmarangoci@icmpp.ro (N.L.M.); pinteala@icmpp.ro (M.P.); 2 Advanced Center for Research and Development in Experimental Medicine “Prof. Ostin C. Mungiu”, “Grigore T. Popa” University of Medicine and Pharmacy, 700115 Iasi, Romania; 3 CEMHTI (CNRS, UPR 3079), Universite d’Orleans, 45100 Orleans, France

**Keywords:** metal-doped carbon dots, MRI signal, relaxivity, relaxometric parameters

## Abstract

In clinical and preclinical magnetic resonance imaging (MRI), image quality is often limited by intrinsic tissue contrast, so paramagnetic agents are used to amplify relaxation differences and improve lesion detectability. Widely used gadolinium-based contrast agents present recognized drawbacks, stimulating interest in nanoscale platforms with tuneable magnetic and biological properties. This review provides a critical analysis on the use of metal-doped carbon nanodots (C-dots) as MRI contrast candidates. We briefly revisit MRI signal formation, spin–lattice ($T_{1}$) and spin–spin ($T_{2}$) relaxation, and relaxometric parameters $r_{1}$ and $r_{2}$ and outline how pulse-sequence choice favours $T_{1}$- or $T_{2}$-dominant agents. We compare approved small-molecule agents with nanostructured systems, highlighting unmet needs in safety, field-strength dependence, multimodality, and organ-specific imaging. A central focus is how nano- and molecular architectures of metal-doped carbon dots govern $r_{1}$ and $r_{2}$: the metal species and oxidation state, its location within the carbon matrix, surface chemistry and hydration, and the accessibility for proton and water exchange can shift performance toward $T_{1}$ or $T_{2}$. Engineered C-dots with controlled composition and metal dopants have proven to pair improved relaxivity with fluorescence, targeting ligands, or therapeutic payloads. Overall, metal-doped C-dots represent a flexible and potentially safer alternative to classical contrast agents; however, successful clinical translation and market uptake will still require standardized relaxometry at clinical field strengths, scalable and reproducible synthesis, and comprehensive in vivo safety and efficacy validation.

## 1. Introduction

Carbon dots (C-dots) are innovative carbon-based nanomaterials, first described by Xu et al. [1] in 2004 as fluorescent impurities formed during the purification of single-walled carbon nanotubes. These quasi-spherical nanoparticles, with typical dimensions below 10 nm, are characterized by an intense fluorescence, excellent biocompatibility and photostability, and versatile surface functionalization, making them ideal for applications in detection, catalysis, and biomedical imaging [2,3,4,5]. However, depending on the synthetic route, surface chemistry, metal doping, and the measurement method, larger apparent sizes can also be reported in the literature. In this context, recent advances in the field of nanomedicine have led to the investigation of C-dots as potential contrast agents for magnetic resonance imaging (MRI), a non-invasive modality recognized for its high spatial resolution and capability for deep-tissue imaging [6]. Currently, only gadolinium-based contrast agents (GBCAs) are used in clinical MRI, as they form stable chelates with organic ligands, thus limiting the toxicity associated with free gadolinium ions [7]. The eight U.S. Food and Drug Administration (FDA)- and European Medicines Agency (EMA)-approved GBCAs that have received authorization for human use are gadopentetate dimeglumine (Magnevist), gadoterate meglumine (Dotarem), gadoteridol (ProHance), gadodiamide (Omniscan), gadobutrol (Godavist), gadoversetamide (Optimark), gadobenate dimeglumine (MultiHance), gadoxetate disodium (Primovist/Eovist), and gadopiclenol (Vueway) (Table 1) [8,9,10,11].

Contrast agents differ in chemical structure, stability, relaxivity, and clearance mechanisms, factors that determine the specific applicability in medical imaging. Despite clinical efficacy, challenges have arisen regarding long-term stability, manifested by the risk of nephrogenic systemic fibrosis in patients with kidney disease and the accumulation of gadolinium in brain tissue [12,13]. Most GBCAs are monomodal agents, specifically designed to increase $T_{1}$ relaxation time, which restricts their ability to provide extensive diagnostic information. These limitations, together with continuous worries about toxicity, have led to the creation of new compounds that are safer and more flexible [14].

Unlike GBCAs, which mostly influence the $T_{1}$ relaxation time, C-dots can be designed to simultaneously modify both $T_{1}$ and $T_{2}$ relaxation times by surface functionalization and doping with different metallic elements. Recent studies have demonstrated that the integration of paramagnetic ions, including gadolinium (Gd^3+^) [14,15,16,17], manganese (Mn^2+^) [18,19,20], and iron oxide (Fe_3_O_4_) [21,22] into C-dots enhances quantum yield and fluorescence characteristics, as well as longitudinal relaxivity ($T_{1}$), thereby transforming these nanoparticles into strong MRI contrast agents. For example, Gd-doped C-dots have been shown to improve $T_{1}$ contrast and to reduce toxicity by forming stable chelates, decreasing the free gadolinium ions [23,24]. Mn-doped C-dots have been reported as effective contrast agents, providing improved $T_{1}$ contrast and minimizing the risks associated with gadolinium retention [25,26]. Fe-doped C-dots demonstrate considerable promise as $T_{2}$ contrast agents, effectively generating high-quality negative images in MRI [27]. As it can be seen, metal-doped C-dots represent promising candidates for the development of dual-mode imaging agents, integrating both fluorescence and magnetic resonance imaging capabilities within a single nanoplatform [28,29,30]. This synergy can improve real-time imaging in biological applications by providing comprehensive anatomical and functional information, allowing for more precise detection and monitoring [31,32,33].

In this review, we summarize and provide a critical analysis on the use of C-dots as contrast agents. First, we present an overview of MRI principles, highlighting the role of $T_{1}$ and $T_{2}$ relaxation in the process of image formation. Further, we discuss the impact of incorporating various metal ions into carbon dots on their magnetic behaviour, specifically focusing on the modifications to $T_{1}$ and $T_{2}$ relaxivities and the implications for practical applications in imaging. Finally, we present a perspective analysis that examines the benefits, drawbacks, and future developments of C-dots as potential next-generation multimodal contrast agents for MRI.

## 2. Basics of MRI

Understanding the fundamental principles behind MRI is important before looking into the development and practical application of new contrast agents. This includes knowing about the magnetic resonance signal and the relaxation processes ($T_{1}$ and $T_{2}$). A strong knowledge of these processes allows us to understand how different metal dopants can influence the physicochemical, magnetic, and optical properties of a potential contrast agent and therefore their performance in MRI. Therefore, we herein outline the fundamental principles of MRI, which will allow a better understanding on how innovative materials can influence image quality and diagnostic accuracy while minimizing toxicity, paving the way for the design of the next generation of multimodal contrast agents.

MRI is one of the most popular non-invasive diagnostic medical imaging techniques that uses a strong magnetic field and radiofrequency (RF) waves to generate detailed three-dimensional images of the internal structures of the body. Unlike X-rays and CT scans, it does not use ionizing radiation, making it safer for clinical applications. Since its introduction in the 1980s, it has offered superior soft tissue contrast and high spatial resolution, especially for complex structures such as muscles, organs, and cartilage [34]. MRI exploits the nuclear magnetic resonance of ^1^H nuclear spins (proton spins) in tissue. The MR signal is produced when an RF field, applied at (or near) the Larmor frequency, excites the net magnetization arising from proton spins, causing it to deviate from the $B_{0}$ direction and generating transverse magnetization. After the RF pulse, the magnetization relaxes toward equilibrium ($T_{1}$ and $T_{2}∕T_{2}^{*}$ processes), and the time-varying transverse component is detected by the receiver coil. Spatial encoding is achieved with magnetic field gradients and image reconstruction. The contrast in MRI images depends on tissue characteristics like water content and fat distribution, allowing for the detection of conditions such as tumours, inflammation, and ischemia [35,36,37]. The MRI signal is influenced by two relaxation processes: spin–lattice ($T_{1}$) and spin–spin ($T_{2}$) relaxation, which determine the strength of the signal. Understanding these processes is extremely important for clinicians, as it helps in the generation of detailed images for the diagnosis of medical conditions. Clinicians can enhance tissue contrast and accurately differentiate between normal tissues and pathological changes by manipulating MRI parameters, which leads to improved patient care and treatment planning [27,34,38,39].

### 2.1. Magnetic Resonance Signal

The fundamental principle of MRI can be described by a simplified classical model in which spinning nuclei behave like tiny magnets. ^1^H nuclear spins are the primary source of MRI signal because of their high abundance in the human body, largely due to water and fat. In the absence of an external magnetic field, the net magnetization is zero because the proton spins are randomly oriented and their magnetic moments cancel on average [38]. Upon the application of a strong magnetic field ($B_{0}$), these magnetic moments align either parallel (low-energy state) or antiparallel (high-energy state) to the field. A slight excess of proton spins aligns parallel, establishing a net magnetization vector along the direction of $B_{0}$. Aligned proton spins exhibit a motion called precession, where they rotate around the direction of the magnetic field at a specific frequency known as the Larmor frequency. This frequency (f) is calculated using the Larmor equation (Equation (1)):

(1) $$f=\gamma B_{0}/2\pi$$

where f indicates the Larmor frequency (the precession frequency of the nuclear spin magnetic moment around the $B_{0}$ direction), γ represents the gyromagnetic ratio (a nucleus-specific constant), and $B_{0}$ is the external magnetic field strength. This equation indicates how fast nuclear spins (their magnetic moments) precess around the direction of the external magnetic field [40,41].

**Figure 1:**
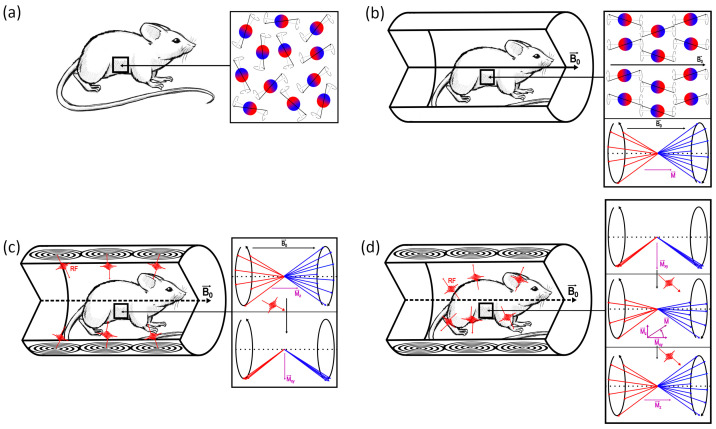
Schematic representation of MRI principle: (**a**) magnetic moments of proton spins from within the body in the absence of an external magnetic field; (**b**) parallel and antiparallel spin-state orientations (and associated magnetic moments) in a strong static magnetic field ($B_{0}$); (**c**) energy absorption from the RF pulse, with transitions between energy levels and phase coherence leading to in-phase precession; (**d**) relaxation phase of proton spins after the RF pulse termination, returning toward their equilibrium state, ($T_{1}$ recovery and $T_{2}∕T_{2}^{*}$ decay) and induction of the detected MR signal in the receiver coil.

As shown in Figure 1, the MRI principle can be summarized as follows. In their equilibrium state, the net magnetization vector of the proton spins aligns with the external magnetic field, producing no measurable signal. At equilibrium, the net magnetization is oriented along $B_{0}$ and does not produce a measurable MR signal because there is no coherent transverse component. A detectable signal is obtained by applying a radiofrequency (RF) field at (or near) the Larmor frequency to excite proton spins and flip the net magnetization away from the $B_{0}$ direction, thereby creating transverse magnetization with phase coherence [42]. During RF excitation, two coupled effects are typically described: (1) energy absorption, where certain protons absorb energy and transition from the low-energy parallel state to the high-energy antiparallel state; and (2) phase coherence, in which the RF pulse synchronizes the precession of the proton spins, causing them to precess in phase with each other. This synchronized precession results in the net magnetization vector, flipping from the longitudinal axis (aligned with $B_{0}$) to the transverse plane, perpendicular to $B_{0}$. The coherent transverse precession at the Larmor frequency generates a voltage in the receiver coils, which constitutes the measured MR signal. Once the RF pulse ends, proton spins undergo relaxation, and the magnetization returns toward its equilibrium state [17,43,44].

### 2.2. Relaxation Processes

Relaxation in MRI refers to the processes by which a nuclear spin returns to its thermal equilibrium state after absorbing radiofrequency energy [34]. It occurs in two forms: longitudinal relaxation ($T_{1}$) and transverse relaxation ($T_{2}$), each characterized by a specific time constant [45].

In the case of longitudinal relaxation, the $T_{1}$ time characterizes the process by which the longitudinal magnetization $M_{z}$ returns to its equilibrium value after the system is excited with a radiofrequency pulse. From a physical point of view, this process is determined by the exchange of energy between the spins and the surrounding environment, which allows the magnetization to gradually recover along the direction of the static magnetic field.

The evolution of longitudinal magnetization is described by one of the solutions of the Bloch equation [46]:

(2) $$M_{z}\left(t\right)=M_{0}\left(1-e^{-t/T_{1}}\right)$$

This relationship shows that the recovery of magnetization follows an exponential recovery law. If we equate time with the process constant ($t=T_{1}$), we obtain the following:

(3) $$M_{z}\left(T_{1}\right)=M_{0}\left(1-e^{-1}\right)≅0.63M_{0}$$

which defines $T_{1}$ as the time required for the longitudinal magnetization to reach approximately 63% of its equilibrium value.

**Figure 2:**
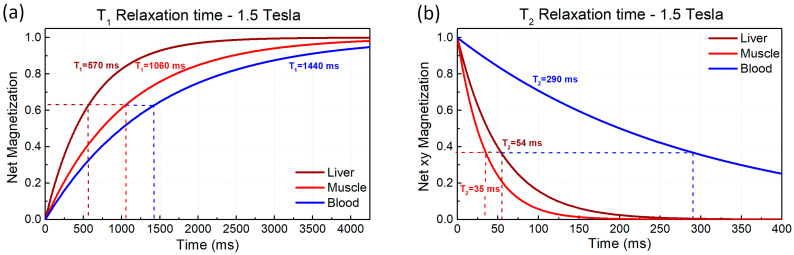
Representative determination of $T_{1}$ and $T_{2}$ relaxation times in a 1.5 T static magnetic field. (**a**) Longitudinal recovery curves of the normalized net magnetization ($M_{z}/M_{0}$) for liver, muscle, and blood, illustrating the definition of $T_{1}$ as the time at which $M_{z}$ reaches $1-e^{-1}\approx 0.63M_{0}$; (**b**) transverse decay curves of the normalized transverse magnetization ($M_{xy}/M_{0}$) for the same tissues, illustrating the definition of $T_{2}$ as the time at which $M_{xy}$ decreases to $e^{-1}\approx 0.37M_{0}$. The indicated $T_{1}$ values are 570, 1060, and 1440 ms, and the corresponding $T_{2}$ values are 54, 35, and 290 ms for liver, muscle, and blood, respectively.

This behaviour is illustrated in Figure 2a, which graphically represents the longitudinal recovery curves ($M_{z}$ as a function of time) and the corresponding characteristic $T_{1}$ values for various tissues [47]. These values reflect the microscopic environment of water proton spins in each tissue, including molecular mobility and interactions with macromolecules. In tissues where these interactions are more effective, such as liver, $T_{1}$ tends to be shorter. On the other hand, in tissues with higher water mobility and weaker interactions, such as blood, $T_{1}$ is longer. As a result, tissues exhibiting prolonged $T_{1}$ times (e.g., water and cerebrospinal fluid) require more time to return to equilibrium, resulting in a darker appearance on $T_{1}$-weighted images. Meanwhile, tissues with shorter $T_{1}$ times (e.g., fat) recover rapidly and thus appear brighter on $T_{1}$-weighted images. Additionally, $T_{1}$ relaxation times also vary depending on the magnetic field strength. At higher field strengths (e.g., 3.0 T vs. 1.5 T), $T_{1}$ relaxation times are generally prolonged, influencing imaging protocols and the interpretation of contrast agents [35,41].

In the case of transverse relaxation, the $T_{2}$ time characterizes the process by which the transverse plane magnetization component, $M_{xy}$, decreases due to the progressive loss of phase coherence between spins. This process is dominated by local spin–spin interactions, which produce microstructural variations in the magnetic field experienced by each individual nucleus. Unlike $T_{1}$ relaxation, $T_{2}$ relaxation does not require net energy transfer to the surrounding lattice; rather, it reflects the rate at which coherent transverse magnetization dephases.

The solution to the Bloch equation that we use to describe the evolution of transverse magnetization is the following:

(4) $$M_{xy}\left(t\right)=M_{0}e^{-t/T_{2}}$$

This relationship shows that transverse relaxation follows an exponential decay, in this case describing an exponential decay. Setting $t=T_{2}$, we obtain the following:

(5) $$M_{xy}\left(T_{2}\right)=M_{0}e^{-1}≅0.37M_{0}$$

which defines $T_{2}$ as the time at which the transverse magnetization decreases to approximately 37% of its initial value.

Figure 2b shows the transverse relaxation curves for the same tissues as in the previous case. As a rule, tissues with longer $T_{2}$ maintain transverse magnetization for a longer time and tend to appear brighter on $T_{2}$-weighted images, whereas tissues with shorter $T_{2}$ lose transverse magnetization rapidly and appear darker. For example, a lower $T_{2}$ in muscle is commonly associated with its organized microstructure and high protein-bound water content, which favours intense spin–spin interactions and faster dephasing. The liver’s more homogeneous microscopic environment facilitates prolonged coherence, whereas in more dynamic environments like blood, phase coherence is generally lost at a slower rate. This means that tissues with long $T_{2}$ values (e.g., water and cerebrospinal fluid) maintain their signal longer and appear bright on $T_{2}$-weighted images, while tissues with short $T_{2}$ values (e.g., muscle, fat, bone marrow) lose their signal quickly and appear dark on $T_{2}$-weighted images.

In an ideal system, all proton spins would experience the same magnetic field and precess at the same Larmor frequency. In practice, the static magnetic field is not perfectly uniform, and additional variations arise from susceptibility differences within tissues. These effects cause proton spins to experience slightly different values of the magnetic field, leading to extra dephasing beyond intrinsic spin–spin relaxation.

The combined effect of spin–spin relaxation and field inhomogeneities is described by the relaxation time $T_{2}^{*}$, called the effective transverse relaxation time, which is always less than or equal to $T_{2}$ [48]. The relationship between the two constants is the following:

(6) $$\frac{1}{T_{2}^{*}}=\frac{1}{T_{2}}+\frac{1}{T_{inh}}$$

where $T_{inh}$ characterizes the contribution of magnetic field inhomogeneity.

The contribution associated with $T_{inh}$ is not intrinsic to the tissue and can be reduced or eliminated by various techniques so that the signal evolution predominantly reflects transverse relaxation. $T_{2}^{*}$ relaxation is always faster than $T_{2}$ because it includes both intrinsic $T_{2}$-relaxation, caused by proton–proton interactions, and magnetic field inhomogeneities, which accelerate dephasing. $T_{2}^{*}$ dephasing can be partially corrected using a 180° refocusing pulse, which realigns the spins and produces a $T_{2}$-signal. However, some information is lost permanently, leading to image artifacts if not properly managed [34,37,49]. To illustrate the time evolution of net magnetization during longitudinal recovery ($T_{1}$) and transverse decay ($T_{2}$), Figure 2a,b show representative relaxation curves for muscle, liver, and blood in a 1.5 T static magnetic field.

## 3. Contrast Enhancement in MRI

MRI is a powerful diagnostic tool renowned for its capacity to produce high-resolution images of soft tissues. However, despite being able to generate detailed tissue contrast, MRI is limited by low sensitivity, which can interfere with the precise differentiation between normal and pathological tissues. To overcome this limitation, contrast agents (CAs) have been developed to enhance image quality by modifying the relaxation properties of surrounding water molecules [50].

Although MRI contrast signal enhancement is predominantly achieved using metal-based contrast agents (e.g., Gd chelates or iron oxide nanoparticles), the use of gadolinium raises concerns regarding complex stability, the potential release of free Gd^3+^, and its possible interaction with tissues. Such interactions may lead to clinically significant adverse outcomes [51].

Given that the retention of metal ions can have considerable adverse effects, metal-free contrast agents have been developed and investigated. Previous reports emphasize that clinical concerns and regulatory considerations related to gadolinium-containing contrast agents have steered research toward safer alternatives capable of providing contrast without the use of metals, while still maintaining relevant imaging performance and an improved biocompatibility profile [51].

Metal-free agents that can be used for MRI may be broadly grouped into four categories: organic radical contrast agents (ORCAs), CEST (chemical exchange saturation transfer) agents, ^19^F-based agents, and hyperpolarized ^13^C (HP^13^C) probes. ORCAs employ stable organic radicals (most commonly TEMPO/PROXYL-type nitroxides) to generate primarily positive T_1_ contrast, whereas CEST produces contrast through frequency-selective saturation and chemical exchange of protons, and ^19^F/HP^13^C agents enable imaging either via non-proton nuclei or through signal amplification rather than via the classical increase in water-proton relaxivity. Beyond these four classes, defect-rich nanodiamonds have emerged as a metal-free approach for MRI contrast enhancement. In particular, detonation nanodiamonds were reported to effectively reduce T1 and yield signal enhancement, attributed to paramagnetic vacancy-type defects in the diamond lattice [52]. Overall, these metal-free paradigms expand the MRI design space by enabling safer formulations and/or distinct mechanistic readouts (molecular exchange, “hot-spot” imaging, or metabolic imaging) [51].

Although there is increasing interest in metal-free MRI contrast approaches, the predominant and clinically established contrast agents continue to be metal-based and use paramagnetic (e.g., Gd^3+^ or Mn^2+^ complexes) or superparamagnetic materials (e.g., iron oxide nanoparticles). These agents enhance image contrast primarily by accelerating the relaxation process of water proton spins, thereby shortening $T_{1}$ and/or $T_{2}$ relaxation times in surrounding tissues. This acceleration results in signal enhancement (hyperintensity) in $T_{1}$-weighted images and signal reduction (hypointensity) in $T_{2}$-weighted images [53,54]. Although most contrast agents affect both $T_{1}$ and $T_{2}$, they are classified as $T_{1}$ or $T_{2}$ contrast agents depending on their predominant effect on water relaxation. In general, the proton spin relaxation time ($T_{i}$), the most importantly water proton spins, can be expressed using Equation (7), where $T_{i0}$ represents the tissue’s inherent relaxation time, and $T_{i}^{CA}$ accounts for the contribution of the contrast agent [55,56].

(7) $$\frac{1}{T_{i}}=\frac{1}{T_{i0}}+\frac{1}{T_{i}^{CA}}\;\left(i=1,2\right)$$

The efficiency of a contrast agent is represented by its longitudinal ($r_{1}$) and transverse ($r_{2}$) relaxivity coefficients which indicate the agent’s ability to increase the relaxation rates of water proton spins in the vicinity as a function of its concentration. Those with an $r_{2}$/$r_{1}$ ratio of less than 5 are generally referred to as $T_{1}$ agents that cause a hyperintense signal on $T_{1}$-weighted images, while the ones having a higher ratio are regarded as $T_{2}$ agents resulting in hypointense signals on $T_{2}$-weighted images. The high longitudinal relaxivity (r_1_) makes the $T_{1}$ relaxation time shorter, which leads to a brighter signal on $T_{1}$-weighted images. CA with high $r_{1}$ values, such as gadolinium-based contrast agents, are usually used for $T_{1}$ imaging. The transverse relaxivity ($r_{2}$) determines how effectively a contrast agent shortens the $T_{2}$ relaxation time, a process that will lead to a darker signal in $T_{2}$-weighted images. An example of CA with high $r_{2}$ values is a superparamagnetic iron oxide nanoparticle (SPION).

MRI contrast agent depends on several key factors, ultimately determining its suitability for specific imaging applications [57]. One of the primary factors is molecular structure and size. The size and structure of a molecule are very important, as a larger molecule or nanoparticle has a higher transverse relaxivity ($r_{2}$), making them particularly effective for $T_{2}$-weighted imaging by enhancing proton spin dephasing. The hydration number (q), which represents the number of water molecules directly coordinated to the contrast agent, influences longitudinal relaxivity ($r_{1}$), improving $T_{1}$ contrast. This effect depends on the accessibility of water molecules to the paramagnetic center and on the efficiency of the water exchange process. Rotational dynamics also influence relaxivity, small molecules being fast-rotating and having higher $r_{1}$ values, thus being appropriate for $T_{1}$ imaging, while larger molecules, rotating more slowly due to limited movements, lead to the increase in $r_{2}$ and therefore are suitable for $T_{2}$ imaging. Moreover, the magnetic characteristics of the CA have had the ability to influence the relaxation rates and strong paramagnetic or superparamagnetic compounds, e.g., a gadolinium-based agent or SPIONs can effectively shorten the relaxation times and thus enhance the contrast. In practical applications, $T_{1}$ contrast agents, characterized by high $r_{1}$ and low $r_{2}$/$r_{1}$ ratios, are commonly used for imaging blood vessels, tumours, and organ function, while $T_{2}$ agents, with high $r_{2}$ and elevated $r_{2}$/$r_{1}$ ratios, are particularly useful for detecting liver lesions, lymph node abnormalities, and iron metabolism disorders [58,59,60]. Understanding these factors is crucial for designing and selecting appropriate contrast agents to optimize MRI diagnostic capabilities.

### 3.1. $T_{1}$-Based Contrast Agents

$T_{1}$-based contrast agents make a significant contribution to MRI imaging enhancement by reducing the longitudinal relaxation time ($T_{1}$) of the water proton spins in the tissue around. Consequently, the signal intensity is elevated, thereby generating hyperintense areas in $T_{1}$-weighted MR images which makes differentiating tissues easier. The most commonly used $T_{1}$ contrast agents are paramagnetic metal complexes, particularly those based on gadolinium (Gd^3+^), due to its seven unpaired electrons in the 4f orbitals, a high magnetic moment, and a long electronic spin relaxation time [61]. However, the “free” or unchelated Gd^3+^ ion is toxic to most biological systems because its ionic radius is similar to that of Ca^2+^, but it carries a higher positive charge. As a result, proteins are unable to distinguish between Gd^3+^ and Ca^2+^ ions, leading free Gd^3+^ to rapidly bind to calcium ion channels and other calcium-dependent proteins. This interference can disrupt biological processes and cause Gd^3+^ to accumulate in organs such as the liver, spleen, and bones [54,62]. In order to reduce this toxicity, a chelation process is needed, and Gd^3+^ must form a stable bond with an organic ligand to create a metal–ligand (ML) complex or chelate. The ligand plays a crucial role by (i) reducing toxicity, (ii) modifying the tissue distribution of the agent, and (iii) affecting the efficiency of Gd^3+^ in shortening $T_{1}$ and $T_{2}$ relaxation times. The common chelating compounds used with Gd are diethylene-triamine-pentaacetic acid (DTPA), 1,4,7,10-tetraazacyclo-dodecane-1,4,7,10-tetra- acetic acid (DOTA), and dipyridoxyl-di-phosphate (DPDP) [63]. The chemical structures of these $T_{1}$ agents are typically characterized by neutral or anionic metal complexes of the form [M(H_2_O)(L)] or [M(H_2_O)(L)]^−^, where M represents a paramagnetic metal ion such as Gd^3+^ or Mn^2+^/Mn^3+^, and Fe^3+^ and L is a macrocyclic or acyclic polyaminopolycarboxylate ligand [8]. This design ensures that Gd remains bound within a safe and stable complex while enhancing its effectiveness as a contrast agent in imaging applications [64].

The mechanistic principle of $T_{1}$ contrast agents is based on their ability to alter the longitudinal relaxation rate (R1), which is defined by the Equation (8):

(8) $$R_{1,obs}=R_{1,tissue}+R_{1,CA}$$

where $R_{1,obs}$ indicates the observed relaxation rate, $R_{1,tissue}$ represents the intrinsic relaxation of the tissue without the contrast agent, and $R_{1,CA}$ is paramagnetic contribution from the contrast agent. Further, the paramagnetic contribution is defined as in Equation (9):

(9) $$R_{1,CA}=r_{1}\cdot \left[CA\right]$$

where $r_{1}$ (mM^−1^·s^−1^) is the longitudinal relaxivity (in mM^−1^·s^−1^), and $\left[CA\right]$ (mM) is the molar concentration of the contrast agent. Using the relationship $R_{1}=1/T_{1}$, this leads to the well-known Equation (10):

(10) $$\frac{1}{T_{1,obs}}=\frac{1}{T_{1,tissue}}+r_{1}\cdot \left[CA\right]$$

This equation demonstrates that the increase in the relaxation rate $R_{1}$ is directly proportional to the concentration of the contrast agent [50]. Therefore, MR image contrast can either be improved by using a contrast agent with a very high relaxivity ($r_{1}$) or by simply increasing the local concentration of the agent. It is important to note that relaxivity, while an internal property of the contrast agent, also depends on factors such as solvent composition and distribution. These parameters may change significantly in vivo, for example, when the contrast agent is restricted to the blood pool or compartmentalized within cells. Therefore, a contrast agent may not affect the relaxation of water proton spins uniformly throughout the tissue; as a result of spatially heterogeneous distribution and microenvironmental effects, the relationship between $R_{1}$ and contrast agent concentration may deviate from linearity [42].

$T_{1}$ contrast agents have proven highly effective in various physiological and pathological conditions such as vascular imaging and to characterize perfusion and microvascular permeability, including brain perfusion with dynamic contrast-enhanced MRI (DCE-MRI), as well as organ-specific functional protocols such as hepatobiliary liver imaging and dynamic contrast-enhanced MRI for renal evaluation. The neural activity mapping is typically performed with BOLD fMRI using endogenous $T_{2}^{*}$ contrast rather than exogenous agents. Their ability to provide positive contrast in DCE imaging makes them indispensable tools for diagnosing and monitoring disease progression. Ongoing research continues to refine these agents, focusing on enhancing their safety, efficiency, and specificity for advanced medical imaging applications [27].

### 3.2. $T_{2}$-Based Contrast Agents

$T_{2}$ contrast agents, or negative contrast agents, are essential in MRI, as they reduce signal intensity in the areas where they are applied. As a result, these agents produce hypointense signals in $T_{2}$- and $T_{2}^{*}$-weighted images, making the affected areas appear darker. This phenomenon takes place as a consequence of the heterogeneity of the magnetic field that surrounds the nanoparticles, which influences the diffusion of water molecules. This diffusion leads to dephasing of the transverse magnetization, ultimately resulting in $T_{2}$ shortening. These CAs are frequently referred to as susceptibility agents because of their strong effects on the local magnetic field. The important thing to know is that the $T_{2}$ shortening effect is a far-off effect, in contrast to $T_{1}$ shortening, which requires a close contact between the water molecules and the $T_{1}$ agents. Moreover, at high magnetic field, the transverse relaxation rate ($R_{2}$) becomes close to a positive constant; thus, it is different from the behaviour of $T_{1}$ contrast agents [65].

Iron oxide nanoparticles, particularly magnetite (Fe_3_O_4_) and maghemite (γ-Fe_2_O_3_), have been widely used as $T_{2}$ contrast agents due to their stability, low toxicity, and strong magnetic properties. Depending on their size, they exhibit either ferromagnetic or superparamagnetic behaviour, with superparamagnetic iron oxide nanoparticles being especially beneficial because they do not retain magnetization once the external field is removed, minimizing aggregation [66]. To prevent unwanted clustering, SPIONs are stabilized using various surface modifications, often involving polymers such as PEG, dextran, chitosan, or polyvinyl alcohol. These coatings influence both relaxivity and biodistribution [67,68]. Based on their size, iron oxide nanoparticles are classified into ultra-small SPIONs (USPIONs, <50 nm), SPIONs (hundreds of nm), and micron-sized iron oxide particles (MPIOs, >1 μm). While USPIONs and SPIONs are administered intravenously, MPIOs are typically used for gastrointestinal imaging [27].

For $T_{2}$ contrast agents, we can also establish the following general relationship (Equation (11)) to describe their effect on the observed transverse relaxation time $T_{2,obs}^{\left(*\right)}$.

(11) $$\frac{1}{T_{2,obs}^{\left(*\right)}}=\frac{1}{T_{2,tissue}^{\left(*\right)}}+r_{2}^{\left(*\right)}\cdot \left[CA\right]$$

Similar to $T_{1}$-shortening agents, the linear relationship between the local decrease in $1∕T_{2}$ and the contrast agent concentration is significantly influenced by the agent’s biodistribution [50].

Despite the advantages of SPIONs, challenges remain, including their inherent negative contrast mechanism, which can sometimes be confused with other pathological conditions and the susceptibility artifacts that may distort images. To overcome these challenges, new imaging methods like off-resonance pulse sequences and inversion recovery ON-resonant water suppression (IRON)-MRI have been developed to enhance the perception of contrast effects without compromising surrounding anatomical detail. Researchers are also exploring alternative magnetic materials to enhance MRI signal sensitivity [68]. For instance, alloy-based nanomaterials and ferrites, created by substituting iron ions with other magnetic atoms like manganese (Mn), zinc (Zn), cobalt (Co), or nickel (Ni), have demonstrated increased saturation magnetization (Ms) and improved transverse relaxivity ($r_{2}$). Manganese-ferrite nanoparticles in particular exhibit high magnetic properties, making them promising candidates as MRI contrast agents. Also, lately, alternative paramagnetic ions, such as dysprosium (Dy^3+^), are being explored as substitutes for iron oxide $T_{2}$ contrast agents in high-field MRI due to their high magnetic moments. Dy^3+^ can be utilized in chelates (e.g., Dy^3+^-DTPA) or as nanoparticles (e.g., Dy_2_O_3_), with studies indicating that their relaxivity significantly increases at higher magnetic field strengths.

While traditional $T_{2}$ contrast agents like SPIONs have significantly contributed to MRI diagnostics, their limitations necessitate ongoing innovation. By developing non-iron oxide nanoparticles, exploring alternative paramagnetic ions, implementing advanced imaging techniques, and creating targeted contrast agents, researchers aim to enhance the effectiveness and reliability of MRI, ultimately leading to improved patient outcomes [8,50].

## 4. Metal-Doped Carbon Dots as MRI Contrast Agents

In recent years, metal-doped carbon dots have emerged as a new class of contrast agents, owing to their unique optical and physicochemical properties and excellent biocompatibility [17,67,68,69,70]. Particularly, doping carbon dots with metals such as Gd^3+^, Mn^2+^, and Fe^2+^ has garnered significant attention in MRI research due to the ability of these metals to shorten relaxation times ($T_{1}$ and $T_{2}$). Such modification leads to higher longitudinal relaxivity ($r_{1}$) values, which improve image contrast and MRI scan quality [71,72]. In this section, we review recent advances in biomedical applications of metal-doped C-dots for MRI, focusing on the role of the metal dopants in modulating the relaxation mechanisms. A summary of the reviewed studies and relevant data from the past five years is provided in Table 2, illustrating how doping with various metals influences relaxivity, contrast enhancement, and overall imaging performance for specific applications [50,73]. This chapter is divided in sections summarizing the application of rare-earth metals, iron, and manganese-doped C-dots, since these are most commonly reported materials for MRI. A final subsection is dedicated to recent trends in co-doping of C-dots with various metals. Data from the literature is critically summarized, emphasizing on the complexity of data comparison due to the lack of consistency in key indicators concerning material characterisation and experimental reporting to describe and understand the MRI potential of metal-doped C-dots.

### 4.1. Rare-Earth Metal-Doped C-Dots

#### 4.1.1. Gadolinium-Doped C-Dots

The unique mechanical, chemical, optical, and magnetic properties of rare-earth metals (REMs) are attributed to the gradual filling of 4f orbitals, resulting in high electron spin magnetic moments, which allows them to improve the signal intensity of magnetic resonance imaging contrast agents by shortening the relaxation period of $T_{1}$-weighted images [12,13,14]. Their large transition numbers, which cover a wide range of wavelengths from the near ultraviolet to the infrared, makes these elements suitable for magnetic detection and as contrast agents with excellent biocompatibility and data storage [11]. The incorporation of rare-earth group metals to C-dots has emerged as an effective strategy in biomedical applications, as it combines the merits of both REMs and C-dots, enabling the “one plus one is greater than two” effect. This has proven to dramatically enhance their luminescent and magneto-optical imaging performances, leading to highly promising practical applications [15,16]. Despite this progress, the field is relatively young, with a limited number of studies, some of which are discussed below.

**Figure 3:**
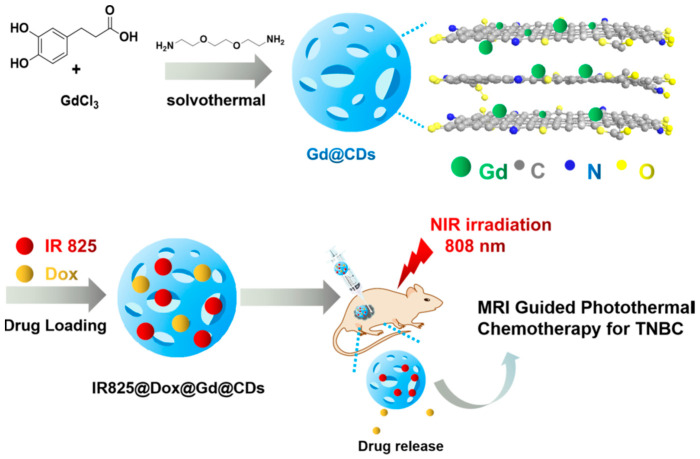
Designing a multifunctional carbon-based nanoplatform using Gd@C-dots for MRI-guided photothermal chemotherapy in triple negative breast cancer (TNBC). Reprinted with permission from Ref. [74]. Copyright Qunjiao Jiang, Li Liu, Qiuying Li, Yi Cao, Dong Chen, Qishi Du, Xiaobo Yang, Dongping Huang, Renjun Pei, Xing Chen, Gang Huang 2021, licensed under a Creative Commons Attribution 4.0 International License.

Jiang et al. [74] synthesized gadolinium-doped carbon dots (Gd@C-dots) with an average size of 2.6 nm, through a hydrothermal method. The obtained Gd@C-dots showed robust fluorescence properties, with a quantum yield of 26.84% for a maximum emission located at 437 nm. In vivo experiments performed with a 1.5 T permanent magnet demonstrated their good performance as MRI contrast, as well as their activity for tumour ablation via NIR laser-induced photothermal therapy and low toxicity. These Gd@C-dots were further integrated into a multifunctional theranostic nanoplatform (Dox@IR825@Gd@C-dots) designed for MRI-guided photothermal chemotherapy targeting triple-negative breast cancer (Figure 3).

Similarly, Li et al. [75] reported a microwave-assisted technique to create Gd-Cdots for MR/FL imaging. Transmission electron microscopy (TEM) analysis revealed that the Gd-Cdots exhibited a uniform spherical morphology with an average hydrodynamic diameter of 6.4 nm. The synthesized Gd-Cdots demonstrated excellent aqueous dispersibility and intense fluorescence with a maximum emission wavelength at 460 nm, maintaining stability in various biological media for at least one month. To assess their MRI performance, the longitudinal relaxivity of 12.85 mM^−1^·s^−1^ were evaluated on a 7 T MRI scanner, with the result being significantly higher than that of commercial Gd-DTPA, enabling enhanced $T_{1}$ contrast imaging. Gd-Cdots show significant potential as an efficient bimodal nanoprobe, enhancing reliability in myocardial infarction diagnosis as well as in imaging-guided surgery and therapy.

Gong et al. [76] also synthesized Gd-Cdots using a microwave-assisted method using sucrose, diethylene glycol, and GdCl_3_ as precursors. TEM revealed spherical nanoparticles of ca. 5 nm in size, with a quantum yield of 5.4% and a maximum emission centred at 521 nm. Relaxivity studies using a 7 T MRI scanner (Bruker Biospin GmbH, Ettlingen, Germany) revealed a very high longitudinal relaxivity of 11.356 mM^−1^·s^−1^, with the $r_{2}$/$r_{1}$ ratio being close to 1, confirming their effectiveness as $T_{1}$-weighted MRI contrast agents. An in vitro cytotoxicity assay on C6 glioma cells demonstrated the good biocompatibility, with more than 90% cell viability. These Gd-Cdots displayed dual-modal imaging capability—fluorescence imaging and $T_{1}$-weighted MRI—for application in glioma cell imaging and other biomedical fields.

Gd@C-dots [77] were also synthesized using a microwave-assisted hydrothermal reaction with β-alanine (β-Ala), ethylenediaminetetraacetic acid (EDTA), and GdCl_3_ as precursors. The obtained amorphous Gd@C-dots displayed a uniform particle size distribution between 7 and 10 nm. The authors reported relaxivity values ($r_{1}$ = 7.92 and $r_{2}$ = 8.98 mM^−1^·s^−1^) comparable and/or higher than those of several commercial GBCA, with very low in vitro cytotoxicity and demonstrated stability over time and across different pH conditions. In addition, in vivo MRI studies of these Gd@C-dots showed intensified $T_{1}$-weighted signals in the liver, spleen, and kidneys, with clearance through the kidneys within 24 h.

In another study, Liao et al. [78] synthesized Gd-doped C-dots using a one-pot hydrothermal method and employing citric acid and GdCl_3_ as precursors. The Gd-Cdots exhibited a longitudinal relaxation rate of 14.08 mM^−1^·s^−1^ and a transverse relaxation rate of 15.85 mM^−1^·s^−1^. Notably, the longitudinal relaxation rate was higher than that of previously reported Gd-Cdots [76]. The cellular toxicity of the synthesized Gd-Cdots was assessed, with half maximum inhibitory concentrations (IC_50_) toward NCI-H446 cells of 6.28 mg/mL, demonstrating the low cytotoxicity. The as-prepared Gd-Cdots were directly applicable to cell imaging and in vivo MR imaging without any additional modification. Similarly, Zheng et al. [79] synthesized Gd-Cdots via a one-step hydrothermal method using gadolinium diethylenetriaminepentaacetic acid and L-arginine as precursors. TEM analysis confirmed a uniform spherical morphology with an average particle size of 5.38 nm. The synthesized Gd-CDs exhibited strong photoluminescence, with the maximum emission peak within the 380–470 nm range and a quantum yield of 57.78%, which enabled fluorescence imaging across multiple spectral channels. To assess their MRI performance, the longitudinal relaxivity ($r_{1}$) of Gd-Cdots was calculated to be 6.27 mM^−1^·s^−1^ from $T_{1}$ measurements acquired on a 3 T MRI scanner, which is significantly higher than that of commercial Gd-DTPA recorded in similar conditions (unfortunately, the study does not provide a rationalization of the better performance of the Gd-Cdots compared to the commercial contrast agent). The authors also reported an excellent biocompatibility, with over 90% viability on A549 cells after 24 h exposure at 250 μg/mL. The in vivo $T_{1}$-weighted MRI of tumour-bearing mice showed that the contrast signals were significantly enhanced as compared to those obtained with Gd-DTPA, thus proving the dual-modal MRI capabilities of their Gd-C-Dots.

Gadolinium-doped carbon dots (AS1411-Gd-Cdots) [16] were synthesized by a solvothermal method combining gadolinium with AS1411 aptamers to enhance targeted fluorescence and MRI for photothermal therapy (PTT). The AS1411-Gd-Cdots displayed a uniform average diameter of 2.5 nm and exhibited strong red fluorescence with a stable emission peak centred at 625 nm. Further characterization revealed a photoluminescence quantum yield of 5.6% and an r_1_ of 13.4 mM^−1^·s^−1^ on a 3 T MRI scanner, confirming their imaging capabilities. In vitro experiments showed that the cancer cells were efficiently targeted and the MR signals in 4T1 cells were significantly brighter than those in the control. These findings position AS1411-Gd-Cdots as effective agents for FL/MR imaging-guided PTT in oncology. Similarly, Gd-Cdots [15] synthesized via a solvothermal method using citric acid, urea, and GdCl_3_ showed an average diameter of 2 nm and exhibited bright red fluorescence with an emission peak at 580 nm. The nanostructures exhibited a high longitudinal relaxivity of 16.0 mM^−1^·s^−1^ using a 1.5 T MRI scanner, making them suitable for $T_{1}$-weighted MRI. The Gd-Cdots exhibited pH-dependent fluorescence and MR sensitivity, functioning as a dual-readout logic system. The biocompatibility was confirmed by in vitro studies on HeLa cells and the in vivo MRI scans which showed enhanced soft tissue contrast. Gd-Cdots are promising for real-time biological monitoring and targeted imaging.

As mentioned above, the field is young and promising, but the comparison of the studies reported in the literature is rather complex, and the mechanism governing the performance of Gd-Cdots in MRI application is far from being well understood. This is mostly attributed to the different experimental conditions and lack of important information concerning the characterization of the materials. Most studies compiled in Table 2 provide only partial datasets concerning key characteristic of the C-dots such as the amount of metal in the final material, the chemical composition and nature of the surface moieties of the C-dots, the concentration of C-dots in the suspensions analysed, etc.

It should be noted that the experimental conditions used to determine MRI performance are often different or not explicitly reported, which is critical since the relaxivity values depend strongly on the magnetic field strength; thus, a comparison can only be conducted under similar magnetic field. In this context, Petrova and co-workers [91] have discussed a mechanistic rationale for field-dependent relaxation by considering how nano-architecture shapes the local magnetic field experienced by surrounding spins. They examined two limiting structural models: (i) a homogeneous distribution of magnetic material throughout a spherical matrix, leading to an approximately uniform internal field with a dipolar field outside the particle, and (ii) a single hard magnetic core embedded in the matrix, producing a dipolar field both inside and outside the granule. Although their study addressed ferromagnetic materials, the key message is general: the spatial distribution of the magnetic component governs the local field and thus the $r_{1}$ and $r_{2}$ behaviour. This framework can also help interpret field-dependent relaxivity in Gd-based nanomaterials. Similarly, the role of the amount of gadolinium incorporated in the C-dots is not always clear, owing to the difficulty in comparing data across studies (when available). For example, Li et al. [75] and Gong et al. [76] report similar $r_{1}$ values recorded at 7 T for materials with comparable Gd loading (~2 at. %), whereas Cardo et al. [77] reported slightly lower $r_{1}$ values (also at 7 T) for a material containing 0.8 at. % of Gd. The same authors also reported lower relaxivity values at 1.5 T (Table 2), almost twice smaller than those reported by Jiao et al. [16] for a material containing ~1.4 at. % of Gd. Altogether, these observations suggest that Gd loading is an important variable that should be analysed in detail, alongside other factors such as particle size, surface chemistry of the carbon dots, and the coordination environment of the metal centres, which can further modulate relaxivity but are not always considered. These considerations emphasize the critical need for comprehensive and standardized reporting protocols to guide a rational design of metal-doped C-dots as next-generation MRI contrast agents.

#### 4.1.2. Other Rare-Earth Metal-Doped C-Dots

Besides gadolinium (Gd), other rare-earth metals such as holmium (Ho), dysprosium (Dy), and neodymium (Nd) have recently been explored as potential alternatives for developing multifunctional MRI contrast agents when doped into carbon dots [80,92,93].

Holmium-doped carbon dots (Ho-Cdots) have been investigated in dual-modal imaging, leveraging the strong paramagnetic properties of Ho^3+^ ions to enhance $T_{2}$-weighted MRI contrast. As an example, Fang et al. [94] synthesized Ho-Cdots with an average size of ~4.5 nm, using DTPA, polyethylenimine, and citric acid as precursors. The optical characterization rendered emission quantum yields (QYs) of up to 8.2% with a fluorescence lifetime of 4.98 ns and a maximum emission peak at 680 nm. Moreover, the prepared Ho-Cdots exhibited significant $T_{1}$-weighted MRI contrast with a longitudinal relaxivity of 2.049 mM^−1^·s^−1^ evaluated using a 1.5 T MRI scanner. In vitro studies on HeLa cells confirmed the excellent biocompatibility (over 80% cell viability), demonstrating minimal cytotoxicity. These findings highlight Ho-CDs as promising candidates for molecular logic-based biosensing, bimodal FL/MR applications, bioimaging, and smart diagnostics.

Dysprosium-doped carbon dots (Dy-Cdots) have been shown to provide superior dark contrast in MRI while maintaining excellent optical properties, making them suitable for multimodal imaging applications. Recently, Atabaev et al. [81] synthesized bifunctional Dy-Cdots via hydrothermal method, with an average size of 12–17 nm. The materials displayed a maximum emission peak at 452 nm (excitation at 363 nm), with a quantum yield of 6.7%. Using a 1.5 T MRI scanner, the transverse relaxivity of the Dy-Cdots was evaluated at 7.42 ± 0.07 mM^−1^·s^−1^, confirming their strong potential as $T_{2}$ contrast agents and their significant potential for dual-modal imaging of living cells.

**Figure 4:**
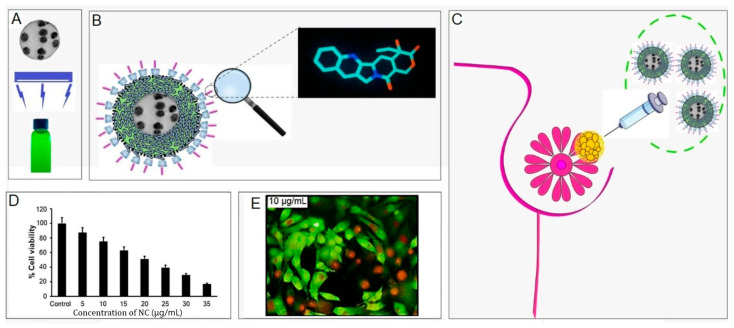
Schematic representation of (**A**) the poly-CD-C-dot: Nd NC; (**B**) loading of CPT in the NC; (**C**) CPT-loaded NC is shot into MCF-7 cells; (**D**) cytotoxicity of the CPT-loaded NC on MCF-7 cells; (**E**) apoptotic response of the cells. Reprinted with permission from Ref. [93]. Copyright 2022, Elsevier.

Neodymium-doped carbon dots (Nd-Cdots) are known for their ability to enhance both $T_{1}$ and $T_{2}$ relaxivities, depending on their structural configuration, which makes them especially good for deep tissue imaging. A recent example by Alexander et al. [93] synthesized a neodymium-doped carbon dot composite with poly-β-cyclodextrin (poly-β-Cdots), with average particle sizes of ~20 nm (Figure 4). The composite exhibited wavelength-dependent photoluminescence, with a maximum emission at 630 nm and paramagnetic properties attributed to the neodymium doping. The Nd-CD/poly-β-Cdots nanocomposite was highly water-soluble and capable of encapsulating the anticancer drug camptothecin (CPT), facilitating a controlled and pH-dependent drug release mechanism. The biological efficacy of the CPT-encapsulated nanocomposite was tested against MCF-7 breast cancer cells, demonstrating enhanced anticancer activity compared to free drug. The luminescence properties of the nanocomposite enabled fluorescence-based tracking of the nanocarrier, while its paramagnetic nature suggested potential applications in MRI-guided drug delivery.

### 4.2. Fe-Doped C-Dots

Over the last few years, iron-based contrast agents have gained significant attention as potential alternatives to gadolinium Gd-based compounds, mainly due to concerns over Gd’s toxicity and its long-term retention in the body. Iron, existing in multiple oxidation states, possesses unpaired electrons that contribute to high electronic spin, allowing it to generate strong local magnetic fields under an external magnetic influence, an essential property for MRI contrast enhancement [95,96,97]. Particularly in biological and medical applications, the relaxation qualities of iron-based contrast agents are determined by the chemical form of iron and how it interacts with its surroundings. Furthermore, Fe-Cdots, as a new type magneto-fluorescent carbon dots, have the advantages of abundant and low-cost metal precursor, high contrast efficiency, excellent biocompatibility, and good optical stability [98,99,100]. A few examples reported in the literature are summarized herein below.

**Figure 5:**
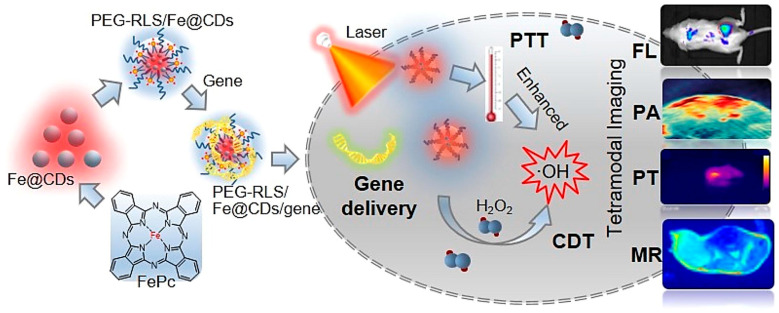
Schematic representation of PEG-RLS/Fe@Cdots as a cancer nanotheranostic platform for photothermal-responsive gene delivery and tetramodal imaging. Reprinted with permission from Ref. [82]. Copyright 2021, Elsevier.

A novel liposomal carbon dot nanohybrid system (PEG-RLS/Fe@Cdots) was developed by Luo et al. [82] as a safe, effective, and photothermally response gene therapy delivery system with multimodal imaging and synergistic tumour therapy (Figure 5). The Fe@Cdots were synthesized via a solvothermal approach using iron (II) phthalocyanine (FePc) as iron source and precursor, followed by modification with an amphiphilic polymer (DSPE-mPEG2000) and a cationic lipopeptide (RLS). The obtained PEG-RLS/Fe@Cdots nanoparticles with an average diameter of ca. 77 nm displayed an $r_{1}$ of 1.25 mM^−1^·s^−1^ (3 T MRI scanner), producing a dose-dependent positive $T_{1}$-weighted MRI contrast enhancement.

In another study, Nimi et al. [83] synthesized citrate-stabilized zerovalent iron nanoparticles (C@ZVI) as a nontoxic, biocompatible, and efficient $T_{1}$ MRI contrast agent for magnetic resonance angiography. These nanoparticles, averaging 10 nm in size, exhibited paramagnetic properties with an $r_{1}$ of 4.93 mM^−1^·s^−1^ recorded using a 3 T MRI scanner, surpassing clinically used gadolinium-based contrast agents (e.g., Dotarem, 3.6 mM^−1^·s^−1^). To enable targeted liver imaging, a multifunctional hybrid nanoconstruct (P@ZVI-Cdots) was developed by modifying C@ZVI with liver-specific polysaccharide pullulan and fluorescent C-dots. While this modification slightly reduced the magnetic properties, P@ZVI-Cdots still exhibited strong fluorescence with an emission maximum at 640 nm and display a promising r_1_ of 3.34 mM^−1^·s^−1^ with a QY of 23%. The $r_{2}$ values were 29.76 mM^−1^·s^−1^ and 19.16 mM^−1^·s^−1^ for C@ZVI and P@ZVI-Cdots, respectively, with $r_{2}$/$r_{1}$ ratios of 6.03 and 5.73, confirming their suitability as $T_{1}$ contrast agents.

For $T_{2}$-weighted imaging, superparamagnetic iron oxide nanoparticles are widely used due to their strong effect on transverse relaxation times. The $T_{2}$-shortening effect of iron oxide doping relies on (i) magnetic field perturbations—SPIONs create local inhomogeneities of the magnetic field, leading to dephasing of water proton spin and a shortened $T_{2}$ relaxation time, ultimately reducing the MRI signal intensity in $T_{2}$-weighted scans, and (ii) particle size and surface coverage. The latest research has revealed that the conglomeration of SPIONs into nanoparticles can lower spin–lattice relaxation ($T_{1}$) while increasing spin–spin relaxation ($T_{2}$), thus making them more efficient as $T_{2}$ contrast agents [97,100].

A recent study by Zhu et al. [84] reported the synthesis of iron-doped carbon dots (TPFe-Cdots) with enhanced imaging and therapeutic capabilities. The prepared TPFe-Cdots exhibited dual-mode fluorescence and MRI properties, along with photodynamic therapy (PDT) functionality. Their nanometric size (~5 nm) was confirmed by TEM, while photoluminescence studies demonstrated an excitation-dependent emission, with a maximum at 450 nm and a quantum yield of 34.49 %. MRI relaxivity analysis revealed that TPFe-Cdots exhibited a $T_{2}$ relaxation rate of 10.388 mM^−1^·s^−1^, indicating their potential as $T_{2}$-weighted contrast agents. In vivo MRI experiments on mouse models showed that TPFe-Cdots selectively accumulated in the thoracic lungs area with scarce distribution in the gastrointestinal tract and tail regions. These results demonstrate that TPFe-Cdots are able to target the chest selectively and thus can be used for imaging pulmonary tissues.

In a recent study, a hydrothermal method was employed by Das et al. [22] to synthesize SPIONs using FeCl_3_ and FeCl_2_ as precursors. TEM images showed spherical nanoparticles of diameter ranging from 40 to 60 nm. A quantum yield of 0.31% was obtained for a maximum emission at 530 nm, which boosts their potential for fluorescence-based imaging applications. When the iron was incorporated in a C-dot matrix, the $r_{2}$ spin–spin rela xivity increased from 29 mM^−1^·s^−1^ (pristine) to 118.3 mM^−1^·s^−1^ (Fe-C-dots), measured with a 1.5 T MRI scanner, improving their performance as $T_{2}$ contrast agents for MRI. In vitro PCR experiments evidenced that mesenchymal stem cells (MSCs) cultured on FeC-dots expressed key gene markers associated with both bone and cartilage differentiation. The facilitated endochondral ossification of the FeC-dots was attributed to their ROS scavenging capacity. Qin et al. [85] also developed FeC-dots using a hydrothermal synthesis method, with ferrous gluconate hydrate and L-aspartic acid as precursors. The obtained FeC-dots showed a homogeneous distribution of particles sizes around 10.3 nm and paramagnetic characteristics with a magnetization value of 0.33 emu·g^−1^. An $r_{2}$ of 9.9 mM^−1^·s^−1^ was determined using a 9.4 T MRI scanner, which is approximately twofold higher than that of a glucose-Fe control (4.5 mM^−1^·s^−1^), together with a significantly high $r_{2}$/$r_{1}$ ratio (76.2). $T_{2}$-weighted MRI studies in U87MG tumour-bearing mice revealed a strong negative contrast effect, which was maximal at 1 h post-injection and dissipated within 4 h. Biocompatibility tests on U87MG and HepG2 cells demonstrated high cell viability (>90%) at concentrations of up to 800 μg·mL^−1^, thus confirming the biosafety of FeC-dots as a multifunctional platform for the in situ detection of bioactive molecules and tumour imaging.

As discussed above for Gd-based systems, the situation is even more challenging for Fe-doped C-dots. The number of studies reporting the MRI applications of Fe-doped C-dots is considerably limited, and the scarcity of comprehensive datasets makes any attempt at comparing studies highly uncertain. In most reports that address Fe-doped C-dots, key parameters such as the actual iron content and/or its chemical state in the final material, the chemical composition and surface functionalization of C-dots, or their concentration used in relaxivity measurements are often missing or only partially provided. This lack of essential data severely prevents a proper understanding of the mechanisms governing the MRI performance of Fe-CDs.

As an example, a deep analysis of the studies by Luo et al. [101] and Nimi et al. [83] has been carried out, as they have measured relaxivity values under similar magnetic field (3 T). Unfortunately, those studies do not report the amount of Fe in the final C-dots, making comparison of the results difficult. If one were to rely solely on the nominal concentrations provided in the synthesis and speculate that both materials contain similar effective Fe concentration, the reported $r_{1}$ values (1.25 and 4.93 mM^−1^·s^−1^, respectively) could be taken to define a tentative relaxivity interval for Fe-doped C-dots under a 3 T field. Within this interval, the variation in relaxivity values would reasonably be attributed to differences in the remaining parameters—such as particle size, surface chemistry, or the coordination environment of Fe. Nevertheless, such a comparison remains uncertain, as none of the relevant parameters are sufficiently documented in the available literature to confirm this interpretation with confidence. This reinforces the critical need for complete, standardized reporting of experimental conditions and material characterization.

### 4.3. Mn-Doped C-Dots

The first paramagnetic agents to be used in vivo for MRI contrast enhancement were manganese-based contrast agents [102,103]. In its bivalent form (Mn^2+^), manganese possesses five unpaired electrons, making it highly effective at shortening the $T_{1}$ relaxation time of water proton spins, thereby increasing the signal intensity in $T_{1}$-weighted MRI images. Additionally, Mn^2+^ exhibits a minor $T_{2}$ effect, causing a reduction in signal intensity and producing dark areas in the images [21,102]. Nevertheless, although Mn^2+^ is generally considered a predominantly $T_{1}$-shortening agent, its relaxivity is dictated less by the metal ion per se and more by the supramolecular environment and proton/water exchange kinetics. In architectures capable of promoting a rapid proton/water exchange (e.g., OH-rich layered structures), Mn-based materials can show enhanced $T_{2}$ effects and even $T_{2}$-dominant contrast [104]. However, at high concentrations, Mn^2+^ can be toxic, necessitating the use of stable, inert complexes to minimize toxicity [22]. For these reasons, incorporating Mn^2+^ in C-dots is a promising strategy to overcome those limitations and further improve their properties, offering potential for safer and more effective MRI contrast agents with multifunctional capabilities [28,29,30,105].

As a few examples, Stepanidenko et al. [19] synthesized manganese-doped carbon dots (Mn-Cdots) via a hydrothermal method using o-phenylenediamine, citric acid and formamide as precursors. TEM confirmed the small size (≤10 nm) of the nanoparticles, while optical characterization revealed broad emissions spanning within the range 400–650 nm. For MRI performance, relaxivity was measured using a 1.5 T MRI scanner and the Mn-Cdots exhibited high $T_{1}$ and $T_{2}$ relaxivities, with $r_{1}$ values ranging from 4.8 to 9.7 mM^−1^·s^−1^ and $r_{2}$ values from 42.2 to 89.0 mM^−1^·s^−1^, depending on the precursor composition and the Mn content. The $r_{2}$/$r_{1}$ ratio of 8.8–10 classified these agents as dual-mode contrast agents suitable for both $T_{1}$ and $T_{2}$ sequences, with an MRI behaviour predominantly characteristic of $T_{2}$ contrast agents [106]. Notably, Mn-Cdots reduced $T_{1}$ relaxation times by up to 6.4% and $T_{2}$ by 42.3%, demonstrating significant contrast-enhancing effects. Their small sizes suggested potential for renal clearance, making them viable candidates for intravenous administration.

Huang et al. [18] synthesized Mn-Cdots via a hydrothermal method using manganese gluconate and L-aspartic acid, exploring their application in contrast-enhanced magnetic resonance imaging (CE-MRI). The Mn-Cdots exhibited small sizes (~5 nm), excellent biocompatibility, and wavelength-dependent photoluminescence in the range of 450–650 nm. A high $r_{1}$ of 10.8 mM^−1^·s^−1^ was evaluated using a 3 T MRI scanner, enabling an effective positive contrast enhancement. In an acute kidney injury model, the Mn-Cdots contrast-enhanced magnetic resonance imaging successfully visualized kidney structure and injury localization, showing alignment with histopathological analysis. Functional renal parameters were derived from MRI data, demonstrating the potentialities of Mn-Cdots as non-Gd contrast agent for kidney imaging. Cytotoxicity and in vivo safety assessments further confirmed their biocompatibility, making Mn-Cdots a promising alternative for non-invasive renal imaging.

Gomez-Blanco et al. [20] synthesized Mn-Cdots nanoclusters (Mn-Cdots-Cs) using ethylenediaminetetraacetic acid, ethylenediamine, and MnCl_2_·4H_2_O as precursors. These nanoclusters of ca. ~150 nm in size displayed a laminar crystalline structure, excellent water solubility, and high fluorescence, characterized by a maximum emission at 413 nm and a quantum yield between 0.17 and 0.20%. MRI studies revealed that Mn-Cdots-Cs had significant $T_{1}$ contrast capability with $r_{1}$ ranging from 2.3 to 3.8 mM^−1^·s^−1^, evaluated using a 1.5 T MRI scanner. Their stability in phosphate buffer facilitated their potential use in biomedical imaging, and the combination of strong fluorescence and MRI contrast pointed out their adequateness as a dual-modal imaging platform for biological applications.

**Figure 6:**
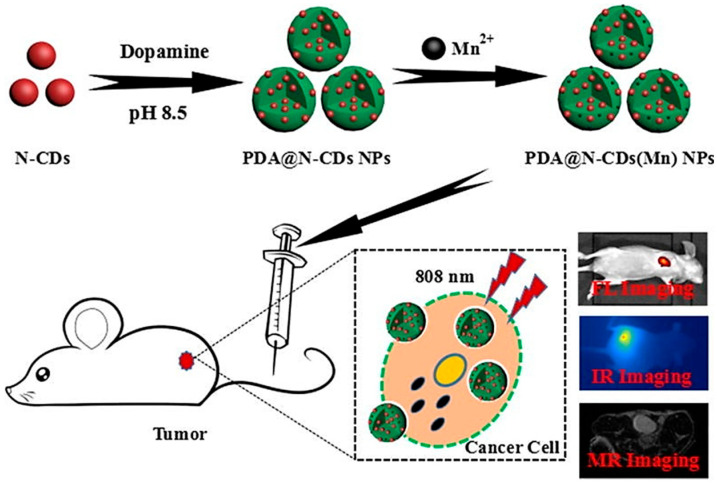
Schematic representation of the synthesis process and mechanism of action of PDA@N-Cdots(Mn) NPs, along with their application in fluorescence, photothermal, and magnetic resonance imaging. Reprinted with permission from Ref. [86]. Copyright 2019, Elsevier.

Manganese-modified polydopamine (PDA) and dual-emissive nitrogen-doped C-dots were synthesized by Zhang et al. [86], yielding PDA@N-Cdots(Mn) nanoparticles with bimodal fluorescence, MRI capabilities, and photothermal activity (Figure 6). The PDA@N-Cdots(Mn) nanoparticles of ca. ~3.2 nm in size exhibited strong near-infrared (NIR) absorbance, high photothermal conversion efficiency (28.2%), and excellent water solubility. Fluorescent imaging was enabled by the N-Cdots, which exhibited red emission at 620 nm and a QY of ca. 4.5%. The presence of Mn^2+^ facilitated MR imaging, producing a bright signal in $T_{1}$-weighted imaging ($T_{1}$, 14.15 mM^−1^·s^−1^) and a darkening effect in $T_{2}$-weighted imaging ($T_{2},$ 39.2 mM^−1^·s^−1^). In vivo experiments confirmed a significant accumulation of the nanoparticles in tumour tissue, with Mn^2+^ contents reaching up to 21.5% of the injected dose, confirming a high tumour-targeting efficiency.

Sun et al. [87] synthesized Mn-Cdots using manganese acetate and o-phenylenediamine as precursors using a one-step solvothermal process. The highly fluorescent nanoparticles obtained (ca. 5 nm) showed maximum emission located at 578 nm and good MRI contrast for dual-modal imaging in cancer diagnosis. The fluorescence intensity of the Mn-Cdots was higher than that of Mn-free CDs, which resulted in better optical properties. The MRI performance showed a high $T_{1}$ relaxivity $r_{1}$ of 12.69 mM^−1^·s^−1^ using a 0.5 T MRI scanner, which was the main reason for the strong positive contrast. In vitro experiments confirmed an excellent cell uptake, low cytotoxicity, and high biocompatibility, while the in vivo study demonstrated the fluorescence and MRI capability.

Comparatively, the studies of Stepanidenko et al. [19] and Gomez-Blanco et al. [20] report materials with similar nominal Mn concentrations, but the resulting relaxivity values differed markedly ($r_{1}$ of 4.8–9.7 mM^−1^·s^−1^ and 2.3–3.8 mM^−1^·s^−1^, respectively). This discrepancy suggests that the nominal loading cannot be used as a reliable indicator, and rather the actual Mn content in the final materials should be provided to explain the observed relaxivities. This highlights the critical importance of fully reporting quantitative information about the metal loading during the synthesis as well as a detailed structural and surface characterization to provide a rational interpretation of the properties-performance correlation of Mn-doped C-dots as MRI contrast agents.

### 4.4. C-Dots Co-Doped with Multiple Metal Ions

Recent advances in co-doping carbon dots with multiple metal ions have significantly enhanced their imaging capabilities by integrating various imaging contrast agents into a single nanoprobe, allowing these agents to complement each other and improve diagnostic and therapeutic outcomes. This multimodal imaging approach combines the high sensitivity of optical imaging with the deep tissue penetration of MRI or CT to achieve more accurate disease detection and monitoring [107]. Additionally, co-doping reduces toxicity, enhances biocompatibility, and amplifies the applications of C-dots for in vivo use, making them promising candidates for advanced diagnostic and therapeutic purposes [108,109,110]. Beyond multimodality, co-doping can also generate synergistic MRI effects by tuning relaxivity-relevant parameters [111,112], including the coordination environment of paramagnetic centres [113], surface hydration and water accessibility, local magnetic susceptibility, and nanoscale dynamics that influence rotational correlation times. As a result, co-doping may enhance $T_{1}$ performance (higher $r_{1}$ and favourable $r_{2}$/$r_{1}$ ratios) or, depending on dopant pairing and architecture, bias contrast toward $T_{2}∕T_{2}^{*}$ through stronger local field inhomogeneities and accelerated dephasing [114,115]. Moreover, rational pairing of ions may support dual-contrast strategies, using one dopant primarily for $T_{1}$ weighted brightening and another to reinforce $T_{2}$-weighted darkening [107,114,116], although the balance is highly dependent on dopant location (lattice versus surface), loading, and water access. Importantly, claims of improved biocompatibility or reduced toxicity in co-doped systems are context-dependent and should be supported by standardized dose-normalized comparisons and long-term clearance data.

**Figure 7:**
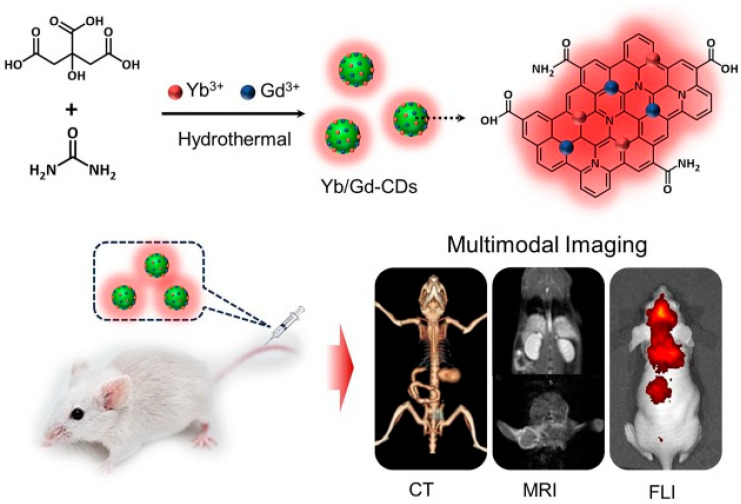
Schematic representation of biocompatible Yb/Gd-Cdots synthesis for in vivo multimodal imaging. Reprinted with permission from Ref. [88]. Copyright 2024, Elsevier.

Shu et al. [88] prepared gadolinium- and ytterbium-doped carbon dots (Yb/Gd-Cdots) through a hydrothermal approach for multimodal fluorescence, MRI, and computed tomography (Figure 7). In the synthesis, citric acid and urea were used as carbon sources and Gd^3+^ and Yb^3+^ as metallic ions to be incorporated into the carbon matrix of the C-dots. The prepared Yb/Gd-Cdots showed an average size of about 5 nm and displayed bright blue fluorescence (emission maximum at 460 nm) with a quantum yield of 14.2%. MRI evaluation revealed a high $T_{1}$ relaxivity value ($r_{1}$ = 11.16 mM^−1^·s^−1^), about 2.8 times higher than the one clinically reported for Gd-DTPA contrast agent (ca. 4 mM^−1^·s^−1^), both obtained at 3 T magnetic field. The ultra-low $r_{2}$/$r_{1}$ ratio of 1.08 further pointed out the capabilities of the prepared Yb/Gd-Cdots as an effective $T_{1}$-weighted MRI contrast agent. In vivo MRI of Kunming mice demonstrated an immediate brightening effect in kidney and liver regions post-injection, with signal intensities peaking at 10 min and gradually recovering to baseline after 24 h, indicating efficient renal clearance. These findings highlight Yb/Gd-CDs as promising nanoscale contrast agents for multimodal imaging.

In a related study, Zhao et al. [89] synthesized gadolinium- and ytterbium-doped C-dots (Gd/Yb@Cdots) for multimodal imaging applications, integrating MRI, X-ray CT, and fluorescence imaging into a single nanoplatform. A one-step hydrothermal synthesis used Gd^3+^ and Yb^3+^ ions as dopants, resulting in nanoparticles with an average diameter of 5.3 ± 0.9 nm. The obtained Gd/Yb@Cdots demonstrated excitation-dependent emission with a maximum emission at 418 nm and a quantum yield of 16.84%. For MRI applications, the Gd/Yb@Cdots exhibited a high longitudinal relaxivity of 6.65 mM^−1^·s^−1^ at 9.4 T, outperforming the clinically used Gd-DTPA contrast agent (r_1_ = 3.69 mM^−1^·s^−1^). The improved relaxivity was attributed to nanometric dimensions of the C-dot nanoparticles that led to an increase in the surface-to-volume ratio, thus allowing more efficient coordination of water molecules with paramagnetic Gd^3+^ ions. In vivo imaging experiments on mice with tumours revealed that the Gd/Yb@Cdots were capable of delivering a robust contrast enhancement in MRI and CT scans, thereby proving their effectiveness for tumour detection.

In their study, Shi et al. [90] synthesized Gd/Ru-Cdots through a one-step microwave-assisted method for fluorescence and magnetic resonance imaging-guided photodynamic therapy. The synthesis used Ru(dcbpy)_3_Cl_2_, citric acid, polyethyleneimine, and GdCl_3_ as precursors, resulting in monodispersed spherical nanoparticles (~4.2 nm) with excellent water solubility. The obtained Gd/Ru-CDs exhibited strong emissive properties in aqueous environments, with a stable red fluorescence emission peak at ~637 nm and a quantum yield of 29.57%. For MRI applications, $T_{1}$ longitudinal relaxivity ($r_{1}$) measured using a 3 T MR scanner demonstrated a concentration-dependent brightening effect in $T_{1}$-weighted MR images. The Gd/Ru-Cdots exhibited an $r_{1}$ value of 6.38 mM^−1^·s^−1^, clearly exceeding that of the clinical contrast agent Gd-DTPA (4.9 mM^−1^·s^−1^). In vitro and in vivo evaluations demonstrated the low cytotoxicity and excellent biocompatibility of Gd/Ru-Cdots. The nanoprobes enabled successful dual-modal fluorescence/MR imaging in 4T1 tumour-bearing mice. In vivo experiments confirmed their ability to induce light-activated tumour suppression, highlighting their significant potential in precise imaging and guided tumour treatment.

Collectively, these reports support the promise of co-doped carbon dots as versatile nanoprobes; however, the extent to which co-doping yields true synergistic gains in MRI depends on dopant placement and water access, emphasizing the need for standardized, head-to-head benchmarking across comparable architectures.

## 5. Challenges and Future Perspectives

Recent advances in metal-doped carbon dots have underscored their potential as versatile and safer contrast agents for magnetic resonance imaging. Gadolinium-based complexes have long dominated $T_{1}$-weighted imaging because of their favourable relaxometric properties, as summarized in Table 1. However, growing evidence of risks, including nephrogenic systemic fibrosis (NSF) in patients with renal impairment and gadolinium deposition in brain tissues, has raised important safety concerns. Consequently, research has increasingly shifted toward alternative agents, including metal-doped carbon dots, that aim to provide comparable or improved contrast efficiency while offering a more favourable safety profile.

The latest evidence consistently indicates that Gd-doped C-dots can exhibit higher $r_{1}$ values than many commercial Gd chelates, often reaching the 11–16 mM^−1^·s^−1^ range [15,16,75,76,78], surpassing widely used Gd chelates such as Dotarem^®^ (3.6 L·mmol^−1^·s^−1^) and Omniscan™ (4.3 L·mmol^−1^·s^−1^). Likewise, Mn-doped C-dots show $r_{1}$ values in the range of 2–14 mM^−1^·s^−1^ and may also provide significant $T_{2}$ effects (with $r_{2}$ values of up to 40–89 mM^−1^·s^−1^) [19], enabling dual-mode $T_{1}$/$T_{2}$ imaging within the same nanoparticle system. Fe-doped C-dots are particularly promising for $T_{2}$-weighted (negative) contrast, with $r_{2}$ values reported from 9.9 up to 118.3 mM^−1^·s^−1^ [22,83,84,85], depending on particle size, doping level, and surface coating. Co-doping carbon dots with multiple metal ions (Gd/Yb@Cdots) [89] have significantly enhanced their imaging capabilities and demonstrated strong $T_{1}$ relaxivity alongside fluorescence and X-ray attenuation, indicating that they could serve as multimodal (MRI + fluorescence + CT) contrast agents.

Beyond improved relaxivity, biocompatibility is a major advantage of metal-doped C-dots. By confining metal ions within a carbon matrix, researchers can substantially reduce the release of free ions, mitigating toxicity concerns associated with conventional Gd chelates. From a clinical translation perspective, a key potential advantage of metal-doped C-dots over conventional gadolinium-based contrast agents (GBCAs) is safety, particularly in circumstances where cumulative exposure or impaired clearance raises concern. Although GBCAs have an overall favourable safety record, their use has been associated with rare but clinically relevant adverse effects and ongoing debate regarding long-term metal retention and risk stratification in vulnerable populations. In this context, metal-doped C-dots could offer safety benefits through (i) non-gadolinium dopants and/or improved metal stabilization within the nanostructure, (ii) higher relaxivity enabling lower administered doses for comparable contrast, and (iii) the possibility of engineering renal-clearable or biodegradable architectures to reduce long-term tissue residency. At the same time, these presumed advantages must be balanced against nanomaterial-specific uncertainties, including potential RES uptake, immunological effects, and dopant leaching under physiological conditions. The step from promising preclinical data to clinical use will depend on how these materials perform in direct comparisons with standard GBCAs. Beyond relaxivity measured under clinically relevant field strengths, the evidence base should include long-term pharmacokinetics and biodistribution, together with GLP-aligned safety testing that covers acute and repeat-dose toxicity, immunotoxicity, genotoxicity, and renal and hepatic safety. Finally, none of this is meaningful without robust manufacturing and quality controls that demonstrate consistent performance from batch to batch. Multiple in vitro and in vivo studies confirm that well-synthesized C-dots often smaller than 10 nm are efficiently excreted through renal pathways, minimizing long-term retention. Moreover, the abundant functional groups on C-dot surfaces facilitate targeted imaging (via ligands, aptamers, or antibodies) and the integration of therapeutic modalities (photothermal or photodynamic therapy), making them attractive for theranostic applications. With their intrinsic fluorescence, magnetic tunability, and customizable surface chemistry, metal-doped C-dots could become the foundation for next-generation “all-in-one” theranostic agents. By coupling MRI contrast with real-time fluorescence guidance and targeted therapy (e.g., chemotherapy, photothermal therapy), clinicians may achieve more precise diagnosis and intervention. Such multifunctional systems would significantly broaden diagnostic capabilities in oncology, cardiology, and neurology.

Conceptually, this diagnostic–therapeutic coupling has also been illustrated in other MRI-relevant nanoplatforms beyond carbon dots. For example, heavily Gd-doped cerium oxide nanoparticles have been proposed for MRI labelling of stem cells [117], while the ceria host is associated with cytoprotective activity (e.g., antioxidant/ROS-scavenging behaviour), supporting the broader theranostic rationale of combining imaging with a beneficial biological function. These observations suggest that a similar design philosophy could be considered for metal-doped C-dots, where the dopant and carbon-dot surface chemistry may be co-engineered to provide both robust contrast performance and an added therapeutic or protective effect.

Although numerous laboratory protocols exist for the preparation of doped C-dots, most of current studies provide a descriptive analysis of the properties-performance in biomedical applications, with a lack of basic understanding of the main factors governing the optical or magnetic properties of the metal-doped C-dots. This, along with the need for reliable large-scale manufacturing of doped C-dots, represent inherent limitations to push forward the application of C-dots as dual-mode imaging nanoplatforms integrating fluorescence and magnetic resonance imaging capabilities. Regulatory approval will require stringent evidence that these new nanomaterials maintain efficacy and safety comparable to or better than existing gadolinium agents, particularly in sensitive populations with renal insufficiency.

Furthermore, full datasets including a comprehensive reporting of experimental details and characteristics of the material (e.g., composition, surface chemistry, metal loading, particle size, measurement conditions) are essential not only for enabling accurate comparisons and ensuring reproducibility but also for supporting the rational design of new metal-doped C-dots. Only when the key parameters influencing relaxivity are clearly identified and reported, it becomes possible to understand which features drive improved performances, what needs to be optimized, and how structural or chemical modifications affect MRI efficiency. Complete and standardized data thus represent a fundamental step forward in the development of C-dot-based contrast agents, providing a solid starting point, guiding targeted improvements, and allowing researchers to build upon existing knowledge rather than starting from scratch.

## 6. Conclusions

This review summarizes recent advances pushing for the application of metal-doped C-dots as competitive contrast agents with adequate $r_{1}$ and $r_{2}$ relaxivities to deliver robust MRI contrast. Yet, their performance is crucially conditioned to the specifics of synthesis (composition, metal loading/dispersion, particle size, solubility) and the need of benchmarking tests (field strength, temperature, physiological medium, pH, dispersion). Imaging efficacy depends on multiple factors, including the dopant species and oxidation state, on whether the dopant resides in the lattice or at the surface, on surface chemistry (density and accessibility of hydroxyl and carboxyl groups), particle size, and aggregation. Crucially, water accessibility and the kinetics of proton/water exchange, which can steer the contrast toward $T_{1}$ or, in certain architectures, yield a predominant $T_{2}$ effect. Comparability across studies further hinges on field strength, temperature, physiological medium, and pH and on analysis choices such as linear fitting of $R_{1}$ or $R_{2}$ versus concentration. Translation will require rigorous stability and safety characterization, standardized reporting of metal doping and relaxivities at clinical fields, head-to-head benchmarks against approved agents at dose-equivalence, and quantitative in vivo demonstrations in clinically relevant models.

Metal-doped C-dots have evolved from a niche research topic into a concept to a promising class of nanomaterials ready poised to reshape MRI contrast technology. Their performance in $T_{1}$- and/or $T_{2}$-weighted MRI sequences can be adjusted depending on the metal used, allowing for undeniably tuned through metal choice and nano-architectural design, enabling precise contrast customization. Reports of higher relaxivities, dual- or multimodal imaging potential, low toxicity, and facile tailorability for targeted or therapeutic applications suggest that these materials represent a meaningful advance. Moreover, their generally favourable biocompatibility and straightforward functionalization for targeting or theranostics suggest meaningful advantages over conventional gadolinium-based agents. As synthetic techniques mature routes established, scale-up and GMP readiness will improve, enabling large-scale safety. Consequently, these novel CNDs are likely to gain traction in both preclinical research and clinical translation, progressing toward clinical evaluation. Their adoption could offer a safer and more flexible approach to medical imaging pathway for MRI contrast provided that reproducibility, standardization, and comparative clinical value are robustly established.

## Figures and Tables

**Table 1:** Gadolinium-based contrast agents at 1.5 T that have received authorization for human use.

Generic Name	Brand Name	$T_{1}$ Relaxivity (mM^−1^·s^−1^)	$T_{2}$ Relaxivity (mM^−1^·s^−1^)	Biomedical Application
Gadopentetate dimeglumine	Magnevist^®^ (Leverkusen, Germany)	4.1	4.6	General MRI contrast agent
Gadobenate dimeglumine	MultiHance^®^ (Milan, Italy)	6.3	8.7	MRI liver and central nervous system imaging (CNS)
Gadodiamide	Omniscan™ (Chicago, IL, USA)	4.3	5.2	CNS and whole-body MRI
Gadoteric acid	Dotarem^®^ (Villepinte, France)	3.6	4.3	CNS and vascular MRI
Gadoteridol	ProHance^®^ (Monroe Township, NJ, USA)	4.1	5	Brain and spine MRI
Gadobutrol	Gadovist^®^ (Leverkusen, Germany)	5.2	6.1	CNS and vascular imaging
Gadoxetate disodium	Eovist^®^ (Leverkusen, Germany)	6.9	8.7	Liver MRI (hepatobiliary imaging)
Gadopiclenol	Vueway™ (Chicago, IL, USA)	12.8	15.1	High relaxivity MRI for vascular and CNS imaging

**Table 2:** Selected properties of metal-doped carbon dots for MRI contrast enhancement.

Metal-Doped C-Dots	Name	Synthesis	Imaging Techniques	Relaxivity ($r_{1},\;r_{2}$) (mM^−1^·s^−1^)	Field Strength	C-Dot Size (nm)	Maximum Emission Wavelength (nm)	QY (%)	Biological Applications	Ref.
REMs (Gd, Ho, Dy, Nd)	Dox@IR825@Gd@CDs	Hydrothermal	MRI		1.5 T	2.58	437	26.8	Cancer treatment	[74]
	Gd-CDs	Microwave-assisted	MRI/FL	$r_{1}$ = 12.85	7 T	6.42	460	54	Myocardial infarction	[75]
	Gd-CDs	Microwave-assisted	MRI/FL	$r_{1}$ = 11.356	7 T	~5	521	5.4	Glioma cell imaging	[76]
	Gd@CNDs	Microwave-assisted hydrothermal reaction	MRI/FL	$r_{1}$ = 7.92 $r_{2}$ = 8.98 $r_{1}$ = 10.50 $r_{2}$ = 18.08	1.5 T 7 T	7–10	-	-	Drug delivery and targeted imaging	[77]
	Gd-CDs	One-pot hydrothermal	MRI/FL	$r_{1}$ = 14.08 $r_{2}$ = 15.85	-	-	-	-	Cell imaging and in vivo MR imaging	[78]
	Gd-CDs	One-step hydrothermal	MRI/FL	$r_{1}$ = 6.27	3 T	5.38	380–470	57.8	In vitro MR cancer cell imaging and in vivo MR imaging	[79]
	AS1411-Gd-CDs	Solvothermal	MRI/FL	$r_{1}$ = 13.4	3 T	2.5	625	5.6	Phototherapy guided imaging (PTT), tumour monitoring	[16]
	Gd-CDs	Solvothermal	MRI/FL	$r_{1}$ = 16	1.5 T	~2	580	2.3	Dual-readout within biological cells	[15]
	Ho-CDs	One-pot pyrolysis	MRI/FL	$r_{1}$ = 2.049	1.5 T	∼4.5	680	8.2	Bimodal FL/MRI imaging, biosensors for the detection of biological markers	[80]
	Dy-CDs	Hydrothermal	MRI/FL	$r_{2}$ = 7.42	-	12–17	630	6.7	Bimodal FL/MRI imaging	[81]
	Nd-CDs	Hydrothermal	MRI	-		∼20		-	Anticancer drug delivery	
Fe	PEG-RLS/Fe @CDs	Solvothermal	MRI/FL	$r_{1}$ = 1.25	3 T	∼77	-	-	Gene delivery, multimodal and real-time imaging in vivo, PTT/CDT synergistic cancer therapy	[82]
	C@ZVI	Chemical reduction	MRI	$r_{1}$ = 4.93 $r_{2}$ = 29.76	3 T	10	640	-	MR angiography and liver-specific bimodal imaging	[83]
	P@ZVI-Cdts			$r_{1}$ = 3.34 $r_{2}$ = 19.16	-	12	-			
	TPFe-CDs	Solvothermal	MRI/FL	$r_{2}$ = 10.388	-	~5 nm	450	-	Thoracic and pulmonary imaging applications	[84]
	FeCDs	Hydrothermal	MRI	$r_{2}$ = 118.3	1.5 T	40–60	530	0.3	Dual-mode imaging (FL, MRI), oncological therapies	[22]
	Fe-CDs	Hydrothermal	MRI/FL	$r_{2}$ = 9.9	9.4 T	3.8	-	-	Peroxidase-mimic nanozyme applications and T_2_-weighted MRI contrast enhancement	[85]
Mn	Mn-CDs	Hydrothermal	MRI/PL	$r_{1}$ = 4.8–9.7 $r_{2}$ = 42.2–89.0	1.5 T	10	400–650	-	Dual-modal nanoprobes for PL and MR bioimaging	[19]
	Mn-CDs	Hydrothermal	MRI	$r_{1}$ = 10.8	3 T	~5	650	-	Diagnosis of acute kidney injury	[18]
	Mn-CND-Cs	One-step microwave-assisted	MRI/FL	$r_{1}$ = 2.3–3.8	1.5 T	~150	413	0.17–0.20	Dual-modal imaging platform for biological applications	[20]
	PDA@N-CDs(Mn)	Self-polymerization	MRI/FL	$r_{1}$ = 14.15 $r_{2}$ = 39.2	-	3.3	620	-	Multimodal bioimaging applications	[86]
	Mn-CDs	Solvothermal	MRI/FL	$r_{1}$ = 12.69	0.5 T	~5	578	-	Dual-modal imaging in cancer diagnosis	[87]
Co-dopped (CDs)	Yb/Gd-CDs	One-pot hydrothermal	MRI/FL	$r_{1}$ = 11.16	3 T	~5	460	-	Nanoscale contrast agents for multimodal imaging	[88]
	Gd/Yb@CDs	One-step hydrothermal	MRI/FL	$r_{1}$ = 6.65	9.4 T	5.26	418	-	Tumour detection	[89]
	Gd/Ru-CDs	One-step microwave-assisted	MRI/FL	$r_{1}$ = 6.38	3 T	~4.2	637	-	Dual-modal fluorescence/MR imaging of 4T1$T_{1}$ tumour	[90]

## Data Availability

No new data were created or analyzed in this study. Data sharing is not applicable to this article.
